# Genetic variations in genes involved in heparan sulphate biosynthesis are associated with *Plasmodium falciparum *parasitaemia: a familial study in Burkina Faso

**DOI:** 10.1186/1475-2875-11-108

**Published:** 2012-04-04

**Authors:** Alexandre Atkinson, Séverine Garnier, Sarwat Afridi, Francis Fumoux, Pascal Rihet

**Affiliations:** 1 UMR928-TAGC, INSERM, 163 Av de Luminy, Marseille F-13288, France; 2 UMR-MD3, 27 Boulevard Jean Moulin, Marseille F-13385, France; 3 Aix-Marseille University, Marseille F-13288, France

**Keywords:** *HS3ST3A1*, *HS3ST3B1*, Heparan sulphate biosynthesis, *Plasmodium falciparum*, Malaria, Parasitaemia, Family-based association, Genetic interaction

## Abstract

**Background:**

There is accumulating evidence that host heparan sulphate proteoglycans play an important role in the life cycle of *Plasmodium *through their heparan sulphate chains, suggesting that genetic variations in genes involved in heparan sulphate biosynthesis may influence parasitaemia. Interestingly, *Hs3st3a1 *and *Hs3st3b1 *encoding enzymes involved in the biosynthesis of heparan sulphate are located within a chromosomal region linked to *Plasmodium chabaudi *parasitaemia in mice. This suggests that *HS3ST3A1 *and *HS3ST3B1 *may influence *P. falciparum *parasitaemia in humans.

**Methods:**

Polymorphisms within *HS3ST3A1 *and *HS3ST3B1 *were identified in 270 individuals belonging to 44 pedigrees and living in Burkina Faso. Linkage and association between parasitaemia and the polymorphisms were assessed with MERLIN and FBAT. A genetic interaction analysis was also conducted based on the PGMDR approach.

**Results:**

Linkage between *P. falciparum *parasitaemia and the chromosomal region containing *HS3ST3A1 *and *HS3ST3B1 *was detected on the basis of the 20 SNPs identified. In addition, rs28470223 located within the promoter of HS3ST3A1 was associated with *P. falciparum *parasitaemia, whereas the PGMDR analysis revealed a genetic interaction between *HS3ST3A1 *and *HS3ST3B1*. Seventy-three significant multi-locus models were identified after correcting for multiple tests; 37 significant multi-locus models included rs28470223, whereas 38 multi-locus models contained at least one mis-sense mutation within HS3ST3B1.

**Conclusion:**

Genetic variants of *HS3ST3A1 *and *HS3ST3B1 *are associated with *P. falciparum *parasitaemia. This suggests that those variants alter both the function of heparan sulphate proteoglycans and *P. falciparum *parasitaemia.

## Background

*Plasmodium falciparum *malaria is transmitted to humans through the bite of infected female *Anopheles *mosquitoes. Sporozoites that have been injected into the skin migrate from the site of injection, and reach the liver, where they invade hepatocytes and change into merozoites; merozoites penetrate and replicate inside red blood cells. Heparan sulphate proteoglycans (HSPGs) may play an important role in the biology of *Plasmodium *through their carbohydrate chains (heparan sulphate) in both the mammalian host and the vector. *Anopheles *heparan sulphate has been shown to bind circumsporozoite protein (CSP), suggesting a role for the carbohydrate chains within *Anopheles *salivary glands for infection and transmission of the parasite [1]. CSP also interacts with the HSPGs on host liver cells, and this interaction has been shown to determine the choice between migrating through or invading the cell [2]. Highly sulphated HSPGs of hepatocytes activates the rodent malaria parasite *Plasmodium berghei *for invasion, whereas the parasite migrates through cells with low sulphated HSPGs in skin and endothelium [2]. In addition, a *P. falciparum *merozoite antigen (EBA-140) has been shown to bind to red blood cells in a heparan sulphate-manner, whereas soluble heparan sulphate and heparin inhibit the merozoite invasion into red blood cells [3,4]. Finally, heparan sulphate is thought to be a receptor for PfEMP1 expressed on infected red blood cells (iRBC), and to mediate the binding of iRBC on endothelial cells or other red blood cells [4-6].

These observations suggest that the outcome of malaria infection may be influenced by variations in the biosynthesis of heparan sulphate, owing to genetic variations within genes encoding the enzymes involved. These include O-sulphotransferases, which catalyze 2-O, 6-O, or 3-O sulphation [7]. The O-sulphation steps are the last steps of the synthesis of heparan sulphate (HS); the sulphation level is a measure of the completion of this synthesis, and is thought to influence the binding properties and therefore the function of HSPGs [7]. The 3-O-sulphation is thought to be a rare event, whereas the 3-O sulphated HS has been shown to serve as an entry receptor of Herpes Simplex Virus 1 (HSV-1) [8]. Interestingly, whereas only one 2-O sulphotransferase, and one 6-O sulphotransferase are known, seven isoforms of 3-O sulphotransferases have been reported [7,9]. The genes encoding the 3-O sulphotransferases are located in different chromosomal regions except for *HS3ST3A1 *and *HS3ST3B1*, and the only significant sequence homology between these proteins occurs in the sulphotransferase domains. Nevertheless, *HS3ST3A1 *and *HS3ST3B1*, which encode the 3-O sulphotransferases 3-OST-3A1 and 3-OST-3B1 respectively, are 700 kb apart in the same chromosomal region, and show a high sequence identity [10]. 3-OST-3B1, which has a sulphotransferase domain 99.2% identical to that of 3-OST-3A1, sulphates an identical disaccharide [11]. Recently, *HS3ST3A1 *that encodes 3-OST-3A1 has been associated with mother-to-child transmission of human immunodeficiency virus (HIV) through a genome-wide association study [12], whereas HSPGs promotes HIV penetration through endothelial cells in a heparan sulphate-manner [13]. This supports the hypothesis that genetic variations within the genes encoding 3-O-sulphotransferases may affect the susceptibility to infectious diseases, such as malaria.

There is a growing body of evidence for human genetic factors controlling the outcome of infection. Familial aggregation and segregation analyses showed the existence of a genetic component of phenotypes related to *P. falciparum *malaria resistance or susceptibility [14,15]. Several candidate genes have been associated with resistance against severe malaria [16]. Linkage or association analyses mapped various loci controlling mild malaria and/or parasitaemia in humans [15,17-19]. The first chromosomal regions that showed linkage to mild malaria and/or parasitaemia were 6p21.3 and 5q31-q33 [15,17,20,21]. However, a limited number of confirmed alleles involved in human malaria have been identified [22,23]. In crosses between genetically defined strains of mice, chromosomal regions responsible for the genetic variance of complex traits can be mapped as quantitative trait loci (QTL) in experimental populations available for precise study under defined conditions. Linkage analyses based on experimental crosses have been done in mice, leading to the mapping of loci controlling *Plasmodium chabaudi *parasitaemia (*Char1-10*) or cerebral malaria [22,24,25]. Notably, such analyses mapped two loci on chromosomes 17 (*Char3*) and 11 (*Char8*), which show extensive conservation of synteny with human chromosomes 6p21.3 and 5q31-q33, respectively [26,27]. The 95% confidence interval of Char8 (chromosome 11 between D11Mit231 and D11Mit30) contained mostly genes, the orthologs of which are located in human chromosome 5q31-q33. Nevertheless, it also contained genes, the orthologs of which are located in human chromosome 17; these include *Hs3st3a1 *and *Hs3st3b1*, which encode 3-O sulphotransferases in mice [26,27], suggesting that genetic variations within *HS3ST3A1 *and *HS3ST3B1 *may influence parasitaemia in humans. This hypothesis was further supported by a linkage study based on microsatellite markers in humans (P.Rihet, unpublished data). This prompted us to screen *HS3ST3A1 *and *HS3ST3B1 *in a population living in an endemic area in Burkina Faso to identify polymorphisms, to evaluate their linkage and association with parasitaemia, and gene-gene interactive effects by using the pedigree-based generalized multifactor dimensionality reduction (PGMDR) method [28].

## Methods

### Subjects

The study subjects live in a rural area, Logoforousso, a village to the south-west of Bobo-Dioulasso (Burkina Faso). The population and the area of parasite exposure have been extensively described [29]. Volunteer families were randomly selected from 3,500 inhabitants. Informed consent was then obtained individually from all participants or their parents. The protocol was approved by the national medical authorities of Burkina Faso. Four mosquito capture sites were chosen, and mosquitoes were collected outdoors 4 days each month (during two nights every 2 weeks). The inoculation rate was 230 infective bites per person per year. The study population comprised 270 subjects from 44 pedigrees corresponding to 71 nuclear families. Eighty-seven parents and 183 siblings were available for genotyping and retained for linkage analysis. The mean age of the siblings was 9.9 ± 4.4 (three to 25 years).

### Phenotyping

Parasitaemia was measured as described [29]. During two years, each family in the rural area was visited 28 times. Blood samples were taken from all individuals present, and only asymptomatic *P. falciparum *parasitaemia measurements were considered in this study. The mean number of asymptomatic *P. falciparum *parasitaemia measurements per subject was 14.9 + 8.1 (range one to 28). The parasitaemia was defined as the number of parasitized erythrocytes observed per microlitre in thin blood films. The analysis was conducted on a logarithmic transformation of parasitaemia adjusted for seasonal transmission and for age that showed a significant effect on parasitaemia (P < 10^-4^). The standardized residual was the phenotype used for linkage and association analyses.

### Genotyping

Each subject underwent a venipuncture, and DNA was extracted from mononuclear cells separated by Ficoll-Hypaque density gradient as described [15]. DNA concentration was assessed with a biophotometer (Eppendorf, Le Pecq, France). The DNA samples (n = 270) were subjected to prior whole-genome amplification with the Illustra GenomiPhi V2 DNA Amplification Kit from General Electric Healthcare (GE Healthcare Biosciences, Pittsburgh, PA, USA) before genotyping. To identify mutations in the promoter region, in all the coding regions and the intron-exon border, defined PCR products were sequenced. All primer pairs were designed with the PRIMER 3 program [30]. There is a strong homology between *HS3ST3A1 *coding sequence and that of *HS3ST3B1*, particularly between exon 2 sequences [10]. Therefore, a first set of primers corresponding to non-coding regions that display sequence variation were designed. PCR was performed with a first set of primers leading to a large product, whereas sequencing reaction was performed with additional internal primers (Additional file 1). Before starting the sequencing reaction, the PCR products were purified with the Qiagen QIAquick PCR purification kit (Qiagen, Hilden, Germany) and quantified by 2% agarose gel electrophoresis. Sequencing reaction was performed with the CEQ DTCS kit (Beckman Coulter, Fullerton, CA, USA) and a CEQ 8800 automated fluorescent sequencer (Beckman Coulter).

### Allele frequencies, haplotype reconstruction and linkage disequilibrium analysis

The compatibility with Mendelian inheritance of marker alleles was checked with the FBAT and MERLIN programs [31,32]. All genotypes passed a Mendelian check with the program FBAT. Using MERLIN, improbable recombination events from SNP maps were checked to detect genotyping errors.

Allele frequencies were calculated by gene counting and deviation from Hardy-Weinberg equilibrium was tested using a Chi-2-test with 1 degree of freedom. Haplotypes were generated based on family genotypic data with MERLIN. Pair-wise LD (Linkage Disequilibrium) was calculated with Haploview and graphical overview of linkage disequilibrium (GOLD) [33,34]. LD between pairs of biallelic markers was tested by the r2 statistic, r^2 ^= (p11p22-p12p21)^2^/p1p2q1q2, where p11, p22, p12, and p21 were two-locus haplotype frequencies, and p1, p2, q1, and q2 were allele frequencies. r^2 ^is the standard c^2 ^statistic divided by the number of chromosomes in the sample. It ranges from 0 to 1. When r^2 ^is 1, SNPs are in complete LD.

### Statistical analyses

Multipoint linkage analyses were performed for sibship and half-sibship data using the software package the MERLIN Package [31]. The regression-based procedure was used for quantitative trait linkage analysis [35]. Combined association and linkage analyses of quantitative traits were carried out using the FBAT program and the QTDT program [32,36]. FBAT calculates a Z score and a two-side *P *value based on a normal approximation. Association in the presence of linkage was assessed using the orthogonal model released in the QTDT program [36]. *P *values were calculated using the likelihood-ratio criterion.

Genetic interactions were analysed by using the pedigree-based generalized multifactor dimensionality reduction method (PGMDR) [28]. The PGMDR is a score-based MDR method that uses the same data reduction strategy as does the original MDR method to detect non-linear genetic interactions [37]. Briefly, the informative siblings were randomly divided into nearly 10 nearly equal subsets, and the cross-validation was repeated 10 times. Each time, nine subsets were used as the training set to construct a binary model with high risk and low risk genotype combinations, while the last subset was considered the testing set. The odds ratio, the corresponding 95% confidence interval, and the *P *value were calculated for the training set. The testing set was used to estimate the prediction accuracy of the model, which is the ratio of correct classifications to the total number of instances classified. The non-parametric sign test was used to evaluate the significance of the prediction accuracy.

The false discovery rate (FDR) procedure was performed to account for the multiple tests performed [38]; an FDR of 5% and an FDR of 10% were applied. Multiple test corrections were carried out for all the linkage, association, and interaction analyses.

## Results

### Descriptive analyses

**Figure 1:**
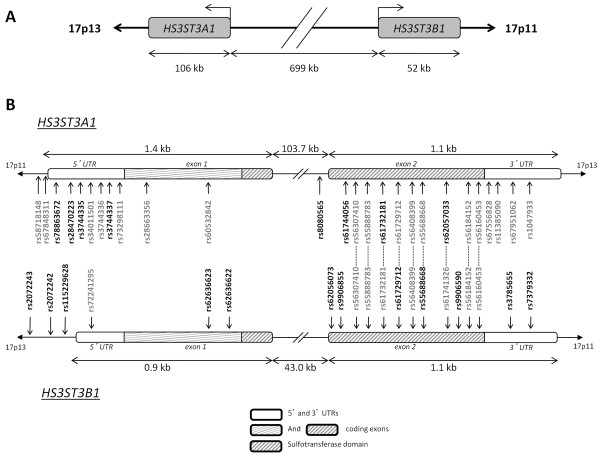
**(A) Diagram of the *HS3ST3A1/HS3ST3B1 *locus**. (B) Location of the known SNPs in relation to the intron/exon structure of *HS3ST3A1 *and *HS3ST3B1*. The SNPs that were detected are in bold. The 5'- and 3'-UTRs are shown by white boxes and the coding exons are shown by hatched boxes. The regions that encode the sulphotransferase domain are shown.

**Figure 2:**
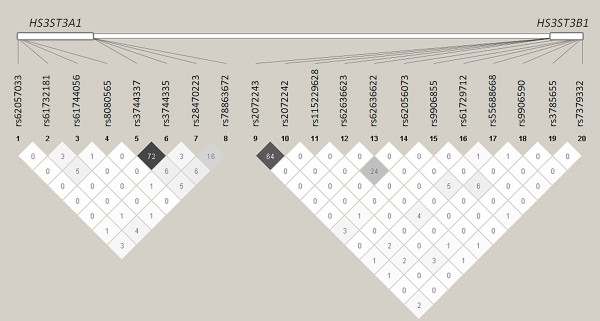
**Pair-wise linkage disequilibrium map within *HS3ST3A1 *and *HS3ST3B1***. Dark denotes highly significant linkage disequilibrium. R^2 ^multiplied by 100 value is shown.

Twenty polymorphisms were identified in the promoter, the exons, the intron-exon junctions, and the 3' untranslated region of *HS3ST3A1 *and *HS3ST3B1 *(Table 1). Notably, some SNPs annotated both in *HS3ST3A1 *and *HS3ST3B1 *were found in either *HS3ST3A1 *or *HS3ST3B1 *(Figure 1). All SNPs passed a Mendelian check, and the detectable genotype errors were < 0.1% based on improbable recombination events from dense SNP maps. There was no deviation from the Hardy-Weinberg equilibrium. Figure 2 shows pair-wise linkage disequilibrium coefficients. There was a significant linkage disequilibrium between some SNPs within either *HS3ST3A1 *or *HS3ST3B1*, whereas no linkage disequilibrium was detected between *HS3ST3A1 *and *HS3ST3B1*. In particular, there was a highly significant linkage disequilibrium between rs3744337 and rs3744335 (r^2 ^= 0.72; *P *< 0.0001) within *HS3ST3A1*; there was also a highly significant linkage disequilibrium between rs2072243 and rs2072242 (r^2 ^= 0.64; P < 0.0001) within *HS3ST3B1*.

**Table 1:** Overview of the polymorphisms genotyped in the African population

SNP Id ^a^	SNP rs#	GRCh37 location (bp)	Localization within gene	Alleles ^b^	MAF ^c^	Molecular change
	rs1047933	13399333	HS3ST3A1 3'UTR	T > A	-	-
	rs67951062	13399487	HS3ST3A1 3'UTR	- > TTT	-	-
	rs11385090	13399495	HS3ST3A1 3'UTR	A > -	-	-
	rs67556828	13399497	HS3ST3A1 3'UTR	- > T	-	-
	rs56160453	13399536	HS3ST3A1 ex2	A > G	-	mis-sense H [His] ⇒ R [Arg]
	rs56184152	13399569	HS3ST3A1 ex2	C > T	-	mis-sense P [Pro] ⇒ L [Leu]
1	rs62057033	13399616	HS3ST3A1 ex2	T > A	0.069	Synonymous
	rs55688668	13399778	HS3ST3A1 ex2	G > A	-	Synonymous
	rs56408399	13399795	HS3ST3A1 ex2	C > T	-	mis-sense R [Arg] ⇒ W [Trp]
	rs61729712	13399884	HS3ST3A1 ex2	G > A	-	mis-sense S [Ser] ⇒ N [Asn]
2	rs61732181	13399928	HS3ST3A1 ex2	G > A	0.043	Synonymous
	rs55888783	13400004	HS3ST3A1 ex2	A > G	-	mis-sense D [Asp] ⇒ G [Gly]
	rs56307410	13400014	HS3ST3A1 ex2	G > A	-	missense V [Val] ⇒ M [Met]
3	rs61744056	13400057	HS3ST3A1 ex2	C > T	0.005	Synonymous
4	rs8080565	13400153	HS3ST3A1 intron	C > T	0.106	-
	rs60532842	13504194	HS3ST3A1 ex1	C > A	-	mis-sense A [Ala] ⇒ S [Ser]
	rs28663356	13504408	HS3ST3A1 ex1	C > A	-	Synonymous
	rs73298111	13504474	HS3ST3A1 5'UTR	C > T	-	-
5	rs3744337	13504665	HS3ST3A1 5'UTR	C > T	0.330	-
	rs3744336	13504689	HS3ST3A1 5'UTR	A > T	-	-
	rs34011501	13504833	HS3ST3A1 5'UTR	- > T	-	-
6	rs3744335	13504884	HS3ST3A1 5'UTR	A > C	0.320	-
7	rs28470223	13505023	HS3ST3A1 5'UTR	C > T	0.161	-
8	rs78863672	13505237	HS3ST3A1 5'UTR	G > T	0.433	-
	rs67848311	13505244	intergenic	C > -	-	-
	rs58718148	13505245	intergenic	C > -	-	-
9	rs2072243	14204380	HS3ST3B1 5'UTR	C > T	0.150	-
10	rs2072242	14204410	HS3ST3B1 5'UTR	T > C	0.184	-
11	rs115229628	14204423	HS3ST3B1 5'UTR	G > A	0.097	-
	rs72241295	14204547-14205186	HS3ST3B1 5'UTR → ex1	639 bp del	-	Large deletion
12	rs62636623	14205082	HS3ST3B1 ex1	G > C	0.020	mis-sense G [Gly] ⇒ R [Arg]
13	rs62636622	14205168	HS3ST3B1 ex1	G > A	0.021	Synonymous
14	rs62056073	14248376	HS3ST3B1 ex2	A > G	0.009	mis-sense I [Ile] ⇒ V [Val]
15	rs9906855	14248423	HS3ST3B1 ex2	C > T	0.173	synonymous
	rs56307410	14248466	HS3ST3B1 ex2	G > A	-	mis-sense V [Val] ⇒ M [Met]
	rs55888783	14248476	HS3ST3B1 ex2	A > G	-	mis-sense D [Asp] ⇒ G [Gly]
	rs61732181	14248552	HS3ST3B1 ex2	G > T/C	-	synonymous
16	rs61729712	14248596	HS3ST3B1 ex2	G > A	0.006	mis-sense S [Ser] ⇒ N [Asn]
	rs56408399	14248685	HS3ST3B1 ex2	C > T	-	mis-sense R [Arg] ⇒ W [Trp]
17	rs55688668	14248702	HS3ST3B1 ex2	G > A	0.066	synonymous
	rs61741326	14248864	HS3ST3B1 ex2	T > A	-	synonymous
18	rs9906590	14248877	HS3ST3B1 ex2	G > A	0.098	mis-sense E [Glu] ⇒ K [Lys]
	rs56184152	14248911	HS3ST3B1 ex2	C > T	-	mis-sense P [Pro] ⇒ L [Leu]
	rs56160453	14248944	HS3ST3B1 ex2	A > G	-	missense H [His] ⇒ R [Arg]
19	rs3785655	14249167	HS3ST3B1 3'UTR	C > T	0.059	-
20	rs7379332	14249433	HS3ST3B1 3'UTR	C > T	0.070	-

^a^Detected SNPs are shown. ^b^Major allele > minor allele. ^c^Allele frequency that was calculated in the study population is shown

### Linkage and association analyses

A regression-based procedure was applied for multipoint linkage analysis using MERLIN. Based on the analysis of the 20 SNPs, parasitaemia was genetically linked to the region containing *HS3ST3A1 *and *HS3ST3B1 *after applying an FDR of 10% (Table 2), with a peak of linkage at rs61729712 (LOD score = 1.207, *P *= 0.009). A linkage was also detected based on the analysis of the 10 SNPs with the highest Minor Allele Frequency (MAF) after applying a FDR of 5% (Table 3); the peak of linkage was, nevertheless, at rs115229628 (LOD score = 1.409, *P *= 0.005).

**Table 2:** Multi-point linkage analyses of parasitaemia to *HS3ST3A1 *and *HS3ST3B1 *based on the 20 SNPs

SNP Id	SNP rs#	LOD	*P *value
1	rs62057033	0.578	0.05
2	rs61732181	0.578	0.05
3	rs61744056	0.610	0.05
4	rs8080565	0.578	0.05
5	rs3744337	0.682	0.04
6	rs3744335	0.634	0.04
7	rs28470223	0.636	0.04
8	rs78863672	0.631	0.04
9	rs2072243	1.111	0.012 ^a^
10	rs2072242	1.106	0.012 ^a^
11	rs115229628	1.144	0.011 ^a^
12	rs62636623	1.138	0.011 ^a^
13	rs62636622	1.140	0.011 ^a^
14	rs62056073	1.136	0.011 ^a^
15	rs9906855	1.157	0.010 ^a^
16	rs61729712	1.207	0.009 ^a^
17	rs55688668	1.199	0.009 ^a^
18	rs9906590	1.199	0.009 ^a^
19	rs3785655	1.200	0.009 ^a^
20	rs7379332	1.201	0.009 ^a^

^a^Significant *P *value after applying an FDR of 10%

**Table 3:** Multi-point linkage analyses of parasitaemia to *HS3ST3A1 *and *HS3ST3B1 *based on the 10 SNPs having the highest MAF

SNP Id	SNP rs#	LOD	*P *value
4	rs8080565	0.548	0.06
5	rs3744337	0.735	0.03
6	rs3744335	0.684	0.04
7	rs28470223	0.749	0.03
8	rs78863672	0.743	0.03
9	rs2072243	1.258	0.008 ^a^
10	rs2072242	1.361	0.006 ^a, b^
11	rs115229628	1.409	0.005 ^a, b^
15	rs9906855	0.684	0.04
18	rs9906590	0.684	0.04

^a^Significant *P *value after applying an FDR of 10%

^b^Significant *P *value after applying an FDR of 5%

Combined linkage and association between each SNP and parasitaemia was further evaluated. FBAT showed evidence of linkage and association between rs28470223 and parasitaemia (*P *= 0.005), whereas the other SNPs were not associated with parasitaemia (*P *> 0.09). The allele C was negatively associated with parasitaemia (Z = -2.77; *P *= 0.005), whereas the allele T was positively associated with parasitaemia (Z = 2.77; *P *= 0.005). This result remained significant after applying an FDR of 5%, and confirmed the linkage signal detected with MERLIN. Furthermore, the linkage was taken into account to test the association between each SNP and parasitaemia by using QTDT; an association in the presence of linkage was detected for rs28470223 (*P <*0.003) after applying an FDR of 5%.

### Interaction analysis

The epistatic effect of *HS3ST3A1 *and *HS3ST3B1 *SNPs was investigated, based on the knowledge of the biological activity of *HS3ST3A1 *and *HS3ST3B1*. Two-, three-, four-, and five-locus models were evaluated. Additional file 2 shows the best models identified on the basis of the classification parameters with the training set (Odds Ratio and *P v*alue) and the cross-validation analysis with the testing set (prediction accuracy and sign test *P *value) after applying a FDR of 5%. The analysis revealed 73 significant multi-locus models, which could be consistently cross-validated (Additional file 2). In other words, the analysis of the training set yielded 73 binary models with high and low risk genotype combinations, which were significantly associated with parasitaemia, and which were validated based on the testing set. All the significant multi-locus models included SNPs located in both *HS3ST3A1 *and *HS3ST3B1*. The significant models included 19 of the 20 SNPs. These include: i) rs3744337, rs3744335, rs28470223, rs78863672, rs2072243, rs2072242, and rs115229628 that are located within the 5'UTR region of either *HS3ST3A1 *or *HS3ST3B1: *ii) rs3785655 and rs7379332 that are within the 3'UTR region of *HS3ST3B1; *iii) rs9906855, rs62057033, rs61732181, rs61744056, rs62636622 and rs55688668 that are synonymous mutations of either *HS3ST3A1 *or *HS3ST3B1*; and iv) rs62636623, rs62056073, rs61729712, and rs9906590 that are mis-sense mutations within *HS3ST3B1*. Interestingly, rs28470223 was in 37 of the 73 significant multi-locus models, and 38 multi-locus models contained at least one mis-sense mutation.

## Discussion

This is apparently the first study to investigate the association between a phenotype related to malaria susceptibility and genes involved in HS biosynthesis. Two genes 700 kb apart, which encode 3-O sulphotransferases involved in the synthesis of HS (*HS3ST3A1 *and *HS3ST3B1*), were considered as candidate genes.

First, *P. falciparum *parasitaemia was found to be genetically linked to *HS3ST3A1 *and *HS3ST3B1 *polymorphisms based on a multipoint linkage analysis. This result is consistent with linkage studies based on microsatellite markers in mice [26,27] and humans (P. Rihet, unpublished data). This suggests that polymorphisms within the chromosomal region may partly explain the variance of parasitaemia, and may affect resistance against malaria. In the same way, rs6503319, which is located in the human chromosomal region genetically linked to parasitaemia (P.Rihet, unpublished data), has been associated with severe malaria [39]. It should be stressed, however, that the location of the peak of linkage depends on the SNPs included in the analysis. This indicates either that the linkage analysis does not accurately locate the causal polymorphisms, or that several polymorphisms within the region may influence parasitaemia.

Second, linkage and association between parasitaemia and rs28470223 was detected. Thus, this one-locus analysis confirmed the linkage signal obtained with the multipoint analysis. Furthermore, rs28470223, which is located within the promoter of *HS3ST3A1*, was associated in the presence of linkage with parasitaemia. This supports the hypothesis that rs28470223 alters both the expression of *HS3ST3A1 *and parasitaemia. Functional studies will be required to evaluate whether rs28470223 affects the binding of a transcription factor, and the level of gene expression. However, it cannot be excluded that rs28470223 is in linkage disequilibrium with the causal polymorphism.

Third, given that *HS3ST3A1 *and *HS3ST3B1 *encode enzymes with a nearly identical activity, a gene-by-gene interaction analysis was conducted based on the PGMDR approach [28]. Two-, three-, four, and five- locus interactions were systematically evaluated. Seventy-three significant multi-locus models, which included SNPs found in both *HS3ST3A1 *and *HS3ST3B1*, were identified. This supports the hypothesis of epistatic interaction between *HS3ST3A1 *and *HS3ST3B1*. In addition, 37 out of the 73 significant multi-locus models included rs28470223 located in the promoter of *HS3ST3A1*, further supporting the hypothesis of a particular role for rs28470223. Other SNPs located in the promoter of either *HS3ST3A1 *or *HS3ST3B1 *and synonymous mutations, which may alter gene expression levels, were also included in several significant multi-locus models. Moreover, 38 multi-locus models contained at least one mis-sense mutation. This suggests a possible functional role of rs62636623, rs62056073, rs61729712, and rs9906590, which alter the sequence of amino acids. rs62056073, rs61729712, and rs9906590 are of major interest because they affect the sulphotransferase domain, suggesting that they may alter the enzymatic activity. Interestingly, site-directed mutagenesis experiments have demonstrated that several amino acid changes in the sulphotransferase domain dramatically reduce the enzymatic activity [40]. In addition, rs62056073, rs61729712, and rs9906590 are close to known mutations that result in the loss of the enzymatic activity [40].

In all, the results suggest that several SNPs within *HS3ST3A1 *and *HS3ST3B1*, the genes encoding 3-OST-3A1 and 3-OST-3B1, may cause variations in either gene expression levels or the enzymatic activity, and that this may result in variations in parasitaemia. Two mechanisms may explain how genetic variations in *HS3ST3A1 *and *HS3ST3B1 *may result in variations in parasitaemia. Genetic variations in *HS3ST3A1 *and *HS3ST3B1 *that alter the 3-O sulphation of HS may affect i) the binding of *P. falciparum *antigen on host cells and/or ii) the pro-inflammatory response.

Together with previous reports [2-4,41], the results suggest that variations in the 3-O sulphation catalyzed by 3-OST-3A1 and 3-OST-3B1 may affect both the binding of *P. falciparum *antigen on host cells and the parasite invasion rate. Highly sulphated HS has been shown to promote a productive invasion of cells by *P. berghei *sporozoites, whereas sporozoites migrate through cells harbouring low sulphated HS [2]. Since the 3-O sulphation is the last step of HS synthesis and occurs after the 2-O and 6-0 sulphation steps, one might assume that highly sulphated HS involved in the sporozoite invasion is 3-O sulphated. This hypothesis is consistent with the data showing that 2-O, 3-O, and N sulphate moieties participate in sporozoite CSP binding [41]. Although the N-sulphation has been shown to be involved in rosette disruption [4-6], and although the O-sulphation has not been reported to influence either the binding of *P. falciparum *antigen on the human erythrocyte surface or the merozoite invasion rate [3], the results showing the inhibition of merozoite invasion by heparin and other highly sulphated glycoconjugates [42,43], and those showing the influence of the O-sulphation on hepatocyte invasion by sporozoites makes this hypothesis relevant.

Genetic variations in *HS3ST3A1 *and *HS3ST3B1 *might also alter the immune response, and more specifically, the inflammatory response. Indeed, pro-inflammatory cytokine and chemokine binding to HS that depends on the sulphation profile of HS controls both the tissue targeting and the local accumulation of cytokines and chemokines [44,45]. Interestingly, *CCR5 *(chemokine (C-C motif) receptor 5) up-regulation was associated with the up-regulation *of HS3ST3A1 *and *HS3ST3B1 *in humans infected by HIV; the authors suggested either that interaction between HS and CCR5 causes up-regulation, or that the promoters of *CCR5*, *HS3ST3A1 *and *HS3ST3B1 *share a cis-regulatory motif binding the same transcription factor [46]. Since *CCR5 *and *HS3ST3A1 *have been associated with HIV infection [12,47], this suggests an interaction at the genetic level affecting resistance to HIV infection. By extension, this suggests that the interaction between immune genes and genes involved in HS biosynthesis may contribute to resistance against other infectious diseases, such as malaria. Additional investigations are needed to evaluate the role of such genetic interactions, and to elucidate how the sulphation profile of HS determines cytokine and chemokine binding.

## Conclusions

This study shows that *HS3ST3A1 *and *HS3ST3B1 *are linked to *P. falciparum *parasitaemia, that rs28470223 within the promoter of *HS3ST3A1 *is associated with *P. falciparum *parasitaemia, and that interactions between *HS3ST3A1 *and *HS3ST3B1 *polymorphisms alter *P. falciparum *parasitaemia. The results also indicate that rs28470223 and four mis-sense mutations within *HS3ST3B1 *strongly contribute to genetic interactions. This study also suggests that other genes involved in HS biosynthesis may affect malaria resistance. In this way, *NDST1 *that plays a major role in HS biosynthesis is located within the chromosome 5q31-q33, which is linked to *P. falciparum *parasitaemia [15].

## Abbreviations

CCR5: Chemokine (C-C motif) receptor 5; CSP: Circumsporozoite protein; GRCh37: Genome Reference Consortium Human genome build 37; HIV: Human immunodeficiency virus; HS: Heparan sulphate; HSV-1: Herpes simplex virus 1; HSPG: Heparan sulphate proteoglycan; LD: Linkage Disequilibrium; MAF: Minor Allele Frequency; iRBC: infected red blood cells; PGMDR: Pedigree-based generalized multifactor dimensionality reduction

## Competing interests

The authors declare that they have no competing interests.

## Authors' contributions

AA carried out the molecular genetic studies, participated in the sequence alignment and performed the statistical analysis. SG participated in the molecular genetic studies and sequence alignment. SA participated in the statistical analysis. FF participated in the design of the study, and revised the results and the manuscript. PR performed the design of the study, supervised the experiments and the statistical analyses, and wrote the manuscript. All authors read and approved the final manuscript.

## Supplementary Material

Additional file 1 **Primer pairs and annealing temperatures used to amplify *HS3ST3A1 *and *HS3ST3B1***. Click here for file

Additional file 2 **The best multi-locus models identified with PGMDR**. All the *P *values were significant after applying a FDR of 5%. Click here for file
