# Supplementary material for: Genetic variations in genes involved in heparan sulphate biosynthesis are associated with Plasmodium falciparum parasitaemia: a familial study in Burkina Faso
Source: Malar J. 2012 Apr 4;11:108. doi: 10.1186/1475-2875-11-108 (PMC3364897; doi:10.1186/1475-2875-11-108)
Supplement: Additional file 1 — Primer pairs and annealing temperatures used to amplify HS3ST3A1 and HS3ST3B1. [file 1475-2875-11-108-S1.DOC]

Additional file 1: Primer pairs and annealing temperatures used to amplify *HS3ST3A1* and *HS3ST3B1*.

|  |  | **PCR and sequencing primers** | | | | | |  |  |  | **Additional internal sequencing primers** | | | |
| --- | --- | --- | --- | --- | --- | --- | --- | --- | --- | --- | --- | --- | --- | --- |
| **Fragment** |  | **Forward (5'-3')** |  | **Reverse (5'-3')** |  | | **PAT (°C)** |  | **AL (bp)** |  | **Forward (5'-3')** |  | | **Reverse (5'-3')** |
| HS3ST3A1 exon 1 |  | ACGTCACCAGCAAGTTTGCTGCCCT | | ACCCCGACAGGTGCCAGAGCATC |  | | 71 |  | 799 |  | - |  | | - |
| HS3ST3A1 exon 2 |  | CTGGCTCCAGCGTGTACTTGAC |  | CACCTCCCAAAGACTCAGTTTTCCT | | 66 | |  | 1292 |  | TTTGTGCTGAAAATATGTTTC | | GAAACATATTTTCAGCACAAA | |
| HS3ST3B1 exon 1 |  | AGAAGCGCCTGTATTTGCAT |  | TACGCCATGTCTCCCTTAGC |  | | 63 |  | 1172 |  | TATATGTTCCTGTACTCGTGC | | GGCGGCTCTTGACATGTTG | |
| HS3ST3B1 exon 2 |  | GAGAGGCGTCACCTTCTGAT |  | AGAAATTTCCCCATGTGCTG |  | | 62 |  | 1229 |  | ATCATCACGGACAAGCACTT |  | | AAGTGCTTGTCCGTGATGAT |

PAT: PCR annealing temp. (°C).

AL: Amplicon length (bp).
